# Prescribing antibiotics: the use of diagnostic tests in general practice. A register-based study

**DOI:** 10.1080/02813432.2021.2004721

**Published:** 2021-11-30

**Authors:** Rikke Vognbjerg Sydenham, Ulrik Stenz Justesen, Malene Plejdrup Hansen, Line Bjørnskov Pedersen, Rune Munck Aabenhus, Sonja Wehberg, Dorte Ejg Jarbøl

**Affiliations:** a Research Unit of General Practice, Institute of Public Health, University of Southern Denmark, Odense, Denmark; b Department of Clinical Microbiology, Odense University Hospital, Odense, Denmark; c Center for General Practice at Aalborg University, Aalborg, Denmark; d Danish Centre for Health Economics, Institute of Public Health, University of Southern Denmark, Odense, Denmark; e Research Unit for General Practice, University of Copenhagen, Copenhagen, Denmark

**Keywords:** Diagnostic methods, drug prescription, epidemiology, family practice, infections

## Abstract

**Objective:**

To assess (i) the pattern of antibiotic prescribing in Danish general practice, (ii) the use of diagnostic tests [point-of-care (POC) and tests analysed at the hospital laboratory (laboratory tests)], and (iii) the frequency of diagnostic testing in relation to antibiotic prescriptions.

**Design:**

Retrospective cross-sectional register-based study.

**Setting:**

General practice in a geographical area of Denmark covering 455,956 inhabitants.

**Subjects:**

We studied redeemed antibiotic prescriptions and performed diagnostic tests in general practice from 2013 to 2017 among inhabitants in nine selected municipalities.

**Main outcome measures:**

Frequency of antibiotic courses. Frequency and type of diagnostic testing performed in relation to types of antibiotics.

**Results:**

A total of 783,252 antibiotic courses were redeemed from general practice with an overall decrease of 19% during 2013–2017. Diagnostic testing increased by 6% during this period. POC tests comprised the majority of performed diagnostic tests (83%) with C-reactive protein (CRP) as the most frequently used test. A 27% increase in the use of laboratory tests was observed. Tests were performed in relation to 43% of all antibiotic courses; most in relation to prescriptions for sulphonamide and trimethoprim (57%) and rarely when prescribing tetracyclines (10%). Conflicting with national guidelines, Danish GPs prescribed fluoroquinolones without performing any kind of diagnostic testing in 48% of the cases.

**Conclusions:**

This study provides an overview of the use of diagnostic tests in relation to antibiotics and creates basis for further research into the variability between types of antibiotics. The study indicates that there is room for improvement to use diagnostic tests as an aid to promote prudent antibiotic use.

**KEY POINTS**
- Diagnostic tests (point-of-care or tests analysed at the hospital laboratory), can increase diagnostic certainty and lead to a reduction in antibiotic use in general practice.
- A decrease in antibiotic courses in general practice in Denmark was observed during 2013–2017, while the use of diagnostic tests increased.
- A diagnostic test was performed in relation to 43% of antibiotic courses.
- Only 52% of prescribed fluoroquinolones was related to a diagnostic test, conflicting with national guidelines.

## Introduction

The World Health Organisation (WHO) considers antimicrobial resistance as one of the largest threats to public health [1]. To reduce the selection of resistant bacteria, it is essential to reduce antibiotic use, especially broad-spectrum antibiotics [2,3]. Refraining from antibiotic prescribing for mild to moderate infections minimises the risk of adverse events and the selection of resistant bacteria.

In Denmark, about 90% of antibiotics are prescribed in the primary health care sector [4], with around 75% issued in general practice [5]. In 2017, the total consumption of systemic antimicrobial agents in Danish primary health care was 14.3 defined daily dose (DDD) per 1000 inhabitant-days (a decline from 15.7 in 2013) [6]. The consumption compares to other Scandinavian countries; Finland 13.6, Sweden 11.3, Norway 14.4, and Iceland 18.8 DDD. The results of a recent Danish study indicate that overuse of antibiotics for respiratory tract infections occurs [7].

Acute infections are common reasons for consulting the general practitioner (GP). Although most infections are of viral origin or non-severe bacterial infections, many of them are treated with antibiotics In Denmark, antibiotics are mainly prescribed for urinary tract infections and acute respiratory tract infections [8–10].

Diagnostic tests are valuable tools for increasing diagnostic certainty to support a prescription of antibiotics or to refrain from this [11,12]. GPs have access to two kinds of tests: (1) Point-of-care (POC) tests that are performed, analysed, and interpreted during a consultation, and (2) laboratory tests that are sent for analysis at a hospital unit and within a few days provide information on microbial aetiology and possibly information on susceptibility. The use of C-reactive protein CRP has been shown in previous studies to improve diagnostic certainty and lead to reduced use of antibiotics [13–15]. However, using diagnostic tests must be applied rationally to reduce unnecessary antibiotic use.

A previous Danish study reported variability in the use of tests between types of antibiotics and between practices [16]. To our knowledge, no studies have explored the use of different types of laboratory tests analysed at hospital facilities in relation to antibiotic prescribing.

The aim of this study was to explore (i) the antibiotic prescribing pattern in Danish general practice, (ii) the use of diagnostic tests (POC or laboratory tests), and (iii) the frequency of use of diagnostic testing in relation to prescribing in total and by type of antibiotic from 2013 to 2017.

## Material and methods

### Study design, setting, and population

This retrospective cross-sectional study aimed at providing a descriptive overview of antibiotic prescribing and the use of diagnostic tests in Danish general practice between 2013 to 2017.

Most Danish citizens are registered with a GP, and services are tax-funded with a mixed capitation and fee-for-service system [17]. The out-of-hours services (OOHS) are organised by GPs in four out of the five regions in the country and in the fifth by the regional health care service. GPs receive a fee for performing POC tests and for drawing and sending samples to hospital laboratory.

This study used national registers as described below. The study sample included redeemed prescriptions for systemic antibacterial drugs in the nine municipalities.

### Data sources and variables

Based on the registers mentioned below, we created a study base consisting of all individuals residing in nine selected municipalities, who at any point between 2003 and 2017 redeemed an antibiotic prescription. As of 1 January 2017, the study base consisted of 405,989 individuals, comprising 89.0% of the total population in the nine municipalities (455,956 inhabitants). The remaining 11% of the population who were not included comprised patients who did not at any point between 2003 and 2017 redeem an antibiotic prescription.

Data were obtained from the following registers and linked through encrypted unique patient and provider identifiers:

1. *The Danish National Prescription Registry*: This database contains complete information on all prescriptions redeemed by residents at outpatient pharmacies. All systemic antibiotics are available only by prescription, and the register is reported as having high validity and completeness [18]. Information about antibiotic ATC codes and the date of redemption was obtained from this registry. All antibacterial drugs were included in the study (ATC-codes J01 and P01AB01) and grouped by type (ATC level 4).
2. *Service Provider Register:* This database contains information on all health service providers and specialty codes. We used specialty codes linked to prescriptions to ensure the inclusion of antibiotic prescriptions and tests exclusively from general practice and out-of-hours services (OOHS).
3. *The Danish National Health Service Register*: This database contains information about activity codes used for reimbursement of services in general practice. Type of service (CRP test, Strep-A, urine dipstick, urine microscopy, urine cultures or susceptibility test in practice laboratory and microscopy of other material) and time of registration were used in the study [19].
4. *The Department of Clinical Microbiology database, Odense University Hospital (DCMO)*: This database contains information on laboratory tests performed in general practice and analysed at the hospital laboratory for the population in the nine municipalities. The database is used for the registration of laboratory test results and for communication to the clinician. The DCMO provided us with information about the type of test (culture skin, culture urine, culture other, respiratory tract bacteria Polymerase Chain Reaction (PCR), *Chlamydia trachomatis*) and the date the test material was received at the DCMO.
5. *The Danish Civil Registration System:* This system contains socio-demographic information about all residents in Denmark. We used information about residency.
6. *Statistikbanken.dk:* A publicly available service from Statistics Denmark, which was accessed to determine population size in the nine municipalities under study for reference use.

For the first and third aims, we created a subsample including all redeemed antibiotic prescriptions from 2013 to 2017 for this population. We restricted the dataset to prescriptions issued exclusively from general practice using the Service Provider Register.

For the second aim (use of diagnostic tests) the dataset included all patients in the study base. Based on The Danish National Health Service Register we included POC tests from general practice from 2013 to 2017. Moreover, we included laboratory tests based on data from The Department of Clinical Microbiology, Odense University Hospital (DCMO*)* (referred to as laboratory tests). Appendix 1 shows a complete list of POC tests and laboratory tests and how they were grouped.

### Analysis and statistics

The antibiotic prescribing pattern was described by computing the redeemed antibiotic courses for each type of antibiotic (ATC level 4) per year and proportions were calculated. If a prescription was preceded by another prescription with the same ATC code within 14 days, it was interpreted as the continuation of a current antibiotic course, and the second prescription was excluded. For the second aim, we computed the frequency of each type of diagnostic test per year and the proportion each type comprised. The third aim (exploring tests performed in relation to the types of antibiotic) was assessed by using the information on performed POC tests and laboratory testing in relation to redeemed antibiotic courses from 2013 to 2017 grouped by ATC codes. Since reimbursements are registered weekly, we linked tests to the Wednesday in the week of registration. A test was determined as linked to an antibiotic prescription if the test was registered from 7 days before to 7 days after an antibiotic prescription was redeemed to account for the fact that diagnostic tests may be used in a variety of ways (to initiate prescribing, ascertain aetiology, follow-up of treatments initiated, etc.) and since there can be a delay between the time of test performance and time of registration.

## Results

### Antibiotic prescriptions from general practice

A total of 783,252 antibiotic courses were redeemed during the study period (2013–2017).

**Figure 1.**
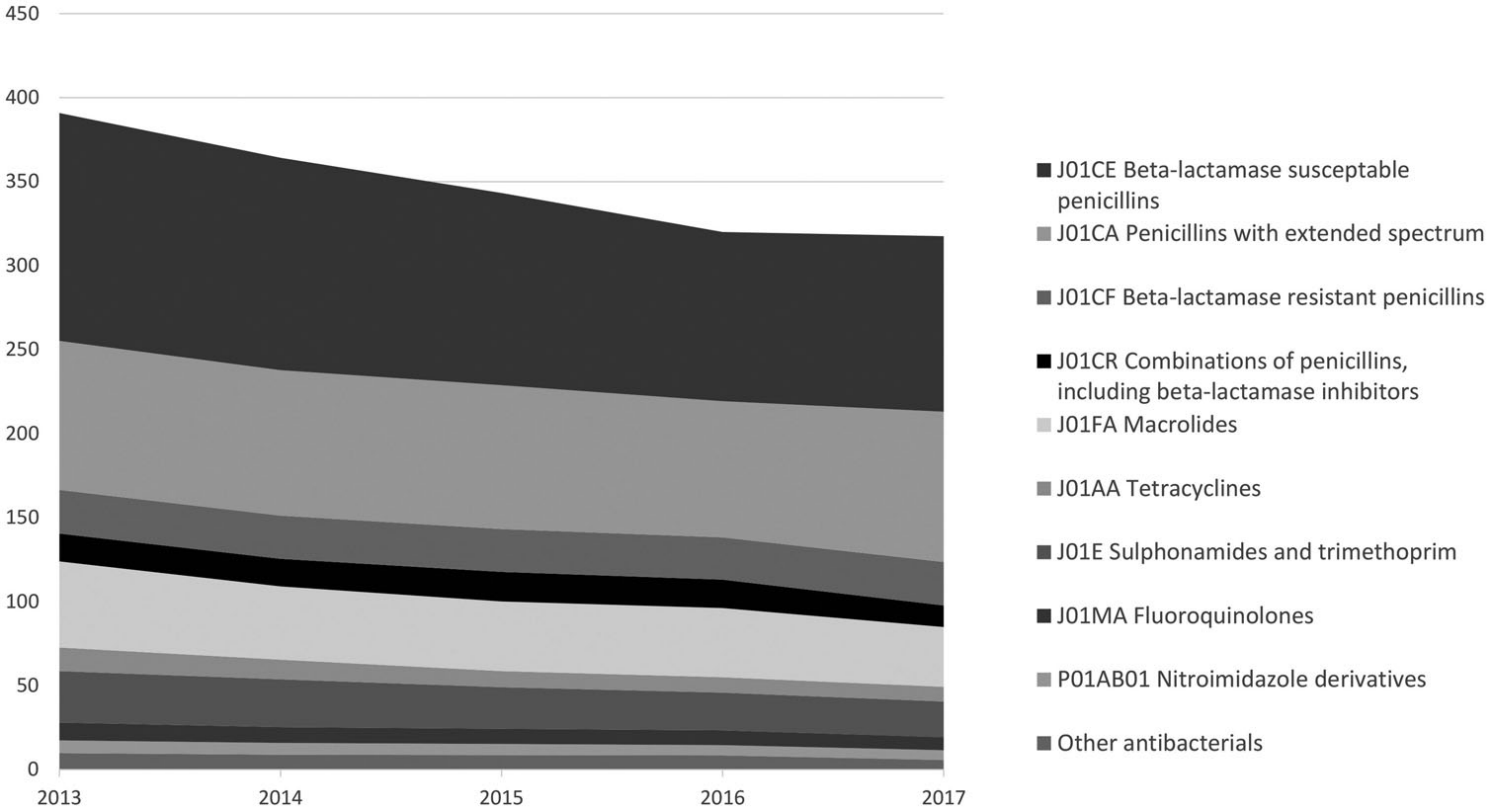
Number of redeemed antibiotic treatments, by type of antibiotic (ATC level 4), issued from general practice per 1000 inhabitants per year from 2013 to 2017 in the nine municipalities under study.

Figure 1 illustrates the redeemed courses per year for each type of antibiotic. In 2013, 391 antibiotic courses were redeemed per 1000 inhabitants decreasing by 19% to 318 per 1000 in 2017. The decrease was observed for all groups of antibiotics except for penicillins with extended-spectrum, where the use remained stable over the five-year period (detailed information is shown in the table in Appendix 2).

### Diagnostic tests performed in general practice

A total of 2,526,634 diagnostic tests were performed during the five years (Table 1). POC tests comprised the majority (83.2%) of tests performed, with CRP and urine tests (dipstick, microscopy, or on-site culture) being the most frequently performed tests. Among the tests analysed at the hospital laboratory, urine cultures were most frequently used.

**Table 1.** Frequency of use of diagnostic tests in 2013–2017 provided as the number of tests per 1,000 individuals per year in total and by type of test.

Tests per 1000 individuals per year	2013	2014	2015	2016	2017	Total tests 2013–2017 *n* (%)
Population in the area	448,149	449,024	450,721	453,561	455,956	
Population in database	410,593	409,712	408,807	407,954	405,989	
Total tests	1179.3	1230.1	1248.6	1265.4	1260.5	2,526,634 (100.0)
Point-of-care tests						
CRP	337.7	363.3	384.2	392.8	398.4	766,586 (30.3)
Strep-A	81.7	83.9	76.5	68.7	68.7	155,094 (6.1)
POC urine tests*	554.7	564.9	561.7	558.2	546.7	1,138,485 (45.1)
Microscopy other	26.6	22.8	19.3	18.1	17.1	42,458 (1.7)
Total POC tests	1000.7	1034.9	1041.6	1037.8	1030.9	2,102,623 (83.2)
Laboratory tests						
Culture skin	25.3	28.8	29.0	30.2	30.8	58,833 (2.3)
Culture urine	79.6	88.1	91.0	97.3	100.0	186,282 (7.2)
Culture other	18.2	20.3	20.7	20.2	21.6	41,227 (1.6)
Respiratory tract bacteria PCR	6.0	7.4	11.4	21.9	19.5	27,017 (1.0)
Chlamydia trachomatis PCR	42.1	42.4	44.0	44.3	43.3	88,288 (3.4)
Laboratory test other	7.5	8.3	10.9	13.7	14.4	22,364 (0.9)
Total laboratory tests	178.6	195.2	207.0	227.7	229.6	424,011 (16.4)

At the far right, the total number of tests performed during the five years are shown with the proportion each test comprises.

*Covering: urine dipstick, microscopy, culture/resistance analysed in practice on-site laboratory.

Table 1 provides an overview of the use of diagnostic tests. The total amount of tests performed in relation to antibiotic prescriptions remained stable during the study period. However, the use of CRP tests increased by 18.0% from 337.7 to 398.4 tests per 1,000 inhabitants from 2013 to 2017, while a decrease was seen for all other types of POC tests. Performing microscopy of other material than urine represented the largest decline (35.6%). Strep-A tests decreased by 15.9% from 81.7 to 68.7 tests per 1,000 inhabitants.

Laboratory tests overall increased by 28,5%. Urine culture was by far the most frequently performed test, followed by testing for *Chlamydia trachomatis*. Cultures comprise the vast majority of laboratory tests compared to PCR tests. However, PCR tests for respiratory tract bacteria have more than tripled (from 6.0 to 19.5 tests per 1,000 inhabitants).

### Diagnostic tests performed in relation to antibiotic prescription

Overall, diagnostic tests were performed in relation to 43.4% of the antibiotic prescriptions (Table 2).

**Table 2.** Testing performed in relation to antibiotic prescription redemption in 2013–2017 (±7 days).

*n* (%)	Any antibiotic	J01CE Beta-lactamase susceptible penicillins	J01CA Penicillins with extended spectrum	J01CF Beta-lactamase resistant penicillins	J01CR Combinations of penicillins	J01FA Macrolides	J01AA Tetracyclines	J01E Sulphonamides and trimethoprim	J01MA Fluoroquinolones	P01AB01 Nitroimidazole derivatives	Other antibacterials
Total prescriptions	783,252 (100.0)	262,334 (100.0)	194,824 (100.0)	57,901 (100.0)	36,231 (100.0)	96,136 (100.0)	24,032 (100.0)	57,510 (100.0)	20,514 (100.0)	14,980 (100.0)	18,790 (100.0)
Any test	339,672 (43.4)	94,340 (36.0)	108,312 (55.6)	21,677 (37.4)	14,801 (40.9)	41,117 (42.8)	2,288 (9.5)	32,657 (56.8)	10,635 (51.8)	7038 (47.0)	6807 (36.2)
Point-of-care tests											
CRP	134,063 (17.1)	62,091 (23.7)	19,452 (10.0)	5,648 (9.8)	13,443 (37.1)	24,131 (25.1)	926 (3.9)	2,823 (4.9)	3,252 (15.9)	1,516 (10.1)	781 (4.2)
Strep-A	37,945 (4.8)	30,296 (11.5)	2,653 (1.4)	214 (0.4)	666 (1.8)	3,537 (3.7)	104 (0.4)	170 (0.3)	81 (0.4)	168 (1.1)	56 (0.3)
POC urine test*	132,741 (16.9)	4,982 (1.9)	79,451 (40.8)	1,100 (1.9)	1,125 (3.1)	3,538 (3.7)	649 (2.7)	27,945 (48.6)	6,598 (32.2)	1,566 (10.5)	5,787 (30.8)
Any point-of-care test	273,996 (35.0)	83,507 (31.8)	91,330 (46.9)	6,688 (11.6)	14,169 (39.1)	28,268 (29.4)	1,557 (6.5)	28,511 (49.6)	8,600 (41.9)	5,275 (35.2)	6,091 (32.4)
Laboratory tests											
Culture skin	26,024 (3.3)	7,167 (2.7)	737 (0.4)	14,020 (24.2)	167 (0.5)	3,100 (3.2)	199 (0.8)	112 (0.2)	354 (1.7)	61 (0.4)	107 (0.6)
Culture urine	73,250 (9.4)	2,495 (1.0)	50,885 (26.1)	475 (0.8)	338 (0.9)	1,224 (1.3)	199 (0.8)	12,144 (21.1)	3,224 (15.7)	492 (3.3)	1,774 (9.4)
Culture other	14,200 (1.8)	4,381 (1.7)	2,005 (1.0)	2,944 (5.1)	596 (1.6)	1,877 (2.0)	164 (0.7)	353 (0.6)	1,158 (5.6)	590 (3.9)	132 (0.7)
Respiratory tract bacteria PCR	8,262 (1.1)	2,548 (1.0)	451 (0.2)	23 (0.0)	427 (1.2)	4,716 (4.9)	26 (0.1)	30 (0.1)	31 (0.2)	10 (0.1)	<5**
Chlamydia trachomatis	17,018 (2.2)	733 (0.3)	2,409 (1.2)	187 (0.3)	14 (0.0)	8,968 (9.3)	649 (2.7)	682 (1.2)	386 (1.9)	2,903 (19.4)	87 (0.5)
Any laboratory test	135,018 (17.2)	17,453 (6.7)	54,905 (28.2)	17,291 (29.9)	1,503 (4.1)	18,995 (19.8)	1,137 (4.7)	12,987 (22.6)	4,909 (23.9)	3,799 (25.4)	2,039 (10.9)

*Covering: Urine dipstick, microscopy, culture/resistance analysed in practice on-site laboratory.

**According to data protection rules exact numbers cannot be reported with <5 individuals.

The most frequently used tests performed in relation to antibiotic prescriptions were CRP (17.1% of redeemed prescriptions) and POC urine tests (16.9%). The CRP test was mainly used in relation to the prescribing of penicillins with beta-lactamase inhibitors (37.1%) followed by macrolides (25.1%). The use of POC urine tests analysed in practice or urine tests sent to the hospital laboratory were frequently related to prescribing of penicillins with extended-spectrum, sulphonamides and trimethoprim, fluoroquinolones, and the group of ‘other antibacterials’.

Sulphonamides and trimethoprim were the antibiotics most often issued in relation to a diagnostic test (56.8% of prescriptions). Penicillins with extended-spectrum followed, with diagnostic tests used in 55.6% of prescribing cases. For fluoroquinolones a test was performed in relation to 51.8% of all prescriptions, with a laboratory test in 23.9% of all the cases. Tetracyclines on the other hand were related to testing in 9.5% of the cases.

## Discussion

### Statement of principal findings

Antibiotic prescribing decreased from 2013 to 2017 for all types of antibiotics except for penicillins with an extended spectrum. Meanwhile, the use of diagnostic tests increased, especially the use of CRP tests, all types of cultures, and PCR for respiratory tract bacteria. This development could be explained by national awareness campaigns from 2016 to 2020 which may influence both GPs and patient expectations. In the same period accreditation in general practice has been mandatory bringing focus on quality in diagnosis and treatment and encouraging comparison of own practice figures (including antibiotic prescribing) with other practices [20].

The study shows that less than half of antibiotic courses had a related diagnostic test, even though Danish GPs have full access to POC tests and laboratory tests and are reimbursed for using these. A large variation was seen between groups of antibiotics with sulphonamides and trimethoprim related to a test in 57% of prescribing cases whereas tetracycline was related to testing in 10% of the cases.

### Strengths and limitations of the study

The study covers an established geographical area with more than 450,000 inhabitants. The study is based on registers that are known to be of very high validity [18]. The Danish National Prescription Registry gives us access to redeemed prescriptions and not to prescriptions that are issued but not redeemed. Previous studies show that primary non-adherence to antibiotics is around 6.5% [21].

We restricted analyses to descriptive statistics on the aggregated level to create an overview of how often and in relation to which type of antibiotic, tests were performed, rather than conducting an in-depth analysis of how the diagnostic tests are used. We found descriptive statistics sufficient to answer our three research questions. An association study on the individual level would provide insights into what patient and GP factors are associated with antibiotics prescribing and testing. This will be a natural next step to pursue. Stratification by type of infection could reveal diagnostic strategies for different types of infection. The databases used for this study provide information about the diagnosis stated on the prescription by the prescriber. However, nearly half of prescriptions lack a disease-specific diagnose [8], and therefore these were not used in this particular study.

Prescriptions from general practice and out-of-hours service (OOHS) cannot be distinguished in the registers. Differences in diagnostic approaches in the two settings must be expected, since fewer diagnostic tools are available in the OOHS, and typically the GP does not know the patient beforehand. GPs in the OOHS had access to a few diagnostic methods (Strep-A, urine dipstick, and CRP) but not to laboratory tests. If the patients are disabled or too ill to go to the GP, the GP will make home visits and have even fewer diagnostic tools.

The study base comprises patients who redeemed at least one antibiotic prescription between 2003 and 2017 (corresponding to 89% of the population in the study area). We do therefore not have information about patients who did not at any point between 2003 and 2017 redeem an antibiotic prescription and cannot report whether they had any tests performed. We cannot test for differences, but individuals with no redeemed antibiotic prescriptions in the 15 years may differ from the rest of the population in terms of being more healthy or less likely to attend the GP. However, we consider that the large study coverage may provide results representative for the population of interest.

The study shows a temporal link between the use of a diagnostic test and antibiotic prescription, but the study cannot confirm that the diagnostic test and antibiotic prescription concern the same health problem. It would be relevant to study the use of tests related to indications for prescriptions but the completeness of specific clinical indications in the registers are only around 68% [8].

### Findings in relation to other studies

Previous studies show that the use of CRP and urine tests can improve diagnostic accuracy and risk classification [15,22–26]. This may in turn lead to a reduction in antibiotic use. Danish national guidelines recommend the use of diagnostic testing to determine that bacteria are the likely cause of disease, thus increasing the probability of a patient benefitting from antibiotic treatment [27]. Danish national guidelines advise to use of diagnostic tests when diagnosing UTIs [28], and we find that recommendations are similar in many other European countries [29–31]. However, extensive testing could increase the risk of overtreatment, as positive tests may indicate the presence of commensals rather than pathogenic bacteria. This is especially the case for UTIs in older women [32]. Also, a Danish study found that GPs who were high-prescribers had higher and possibly excessive use of Strep-A test [33].

PCR tests for respiratory tract infections are not commonly used. However, the prevalence has more than tripled over the study period. Reasons behind this could be increased focus on not prescribing macrolides without diagnostic testing and epidemics of *Bordetella pertussis* and *Mycoplasma pneumonia* [27].

We assessed the use of diagnostic tests from 2013 to 2017 and found higher proportions of prescriptions related to a test compared to a Danish study by Haldrup et al. exploring the same topic also using national registers but from 2004 to 2013 [16]. This may indicate a small increase in the use of tests over time. For fluroquinolones, we found that a test was performed in 52% of prescriptions over the study period 2013–2017, whereas the study from Haldrup et al. found a test performed in only 37% in 2013. For fluoroquinolones, a national antibiotic guideline from 2013 recommends a laboratory test (yielding a susceptibility pattern) before prescribing (a few exceptions were stated for patients allergic to penicillin who have pyelonephritis or exacerbation in chronic obstructive pulmonary disease, patients with severe gastroenteritis at higher risk of complications, and men above 35 years with epididymitis). Although our results show an increase in tests in relation to fluoroquinolones compared to Haldrup et al., still only around half of these prescriptions were related to tests, and not all these tests will yield a susceptibility pattern as specified in recommendations.

Clinical uncertainties are inherent in medicine but more pronounced in primary care, and clinicians may opt for diagnostic testing for (1) a more certain diagnosis; (2) monitoring and susceptibility patterns to ensure correct treatment [34]. However, testing is only indicated in cases with reasonable doubt regarding diagnosis and/or treatment. Whaley et al. found that clinicians expressed diagnostic uncertainty in 43% of visits with antibiotic-appropriate diagnoses corresponding roughly to our findings regarding the use of diagnostic tests and antibiotic prescribing [35].

### Meaning of the study

We found a decreasing use of antibiotics concurrent with an increase in diagnostic testing. This could indicate an increasing awareness of antimicrobial resistance among GPs and efforts to avoid unnecessary antibiotic use.

We found that for sulphonamide and trimethoprim, tests were performed in relation to just 57% of all prescriptions. This group of antibiotics is almost exclusively used for urinary tract infections in Denmark in which guidelines advise active use of diagnostic tests. This could indicate room for improvement since tests are easily available, the GPs are reimbursed for using them, and the tests can assist the GPs in identifying patients who would benefit from antibiotic treatment and, equally important, who would be better off without. In a broader perspective, increased use of these tests may increase diagnostic certainty and reduce the use of antibiotics.

Importantly, access to diagnostic tests is not the same as making good use of them. We do not know the correct cut-off between antibiotic prescription and the use of diagnostic testing. In general, tests are intended to increase diagnostic certainty, provide susceptibility patterns, or to monitor treatment (including wait-and-see) effects. Importantly, many cases of antibiotic prescribing do not necessarily include diagnostic testing.

This study provides knowledge on the extent to which laboratory tests are used. The study cannot determine whether the use of tests is associated with a higher quality of care. To investigate this further, it would be relevant to explore the variation in the use of tests among GPs and for which patients and indications they are used. Furthermore, it is relevant to study if the testing actually makes a difference in the use of antibiotics.

## Appendix 1

List of laboratory tests and groups.

**Table:**

Group	Type	Anatomical localisation	Restrictions
Culture skin	Ulcer—swab		
	Ulcer—swab decubitus ulcer		
	Ulcer swab surgery/cicatrice		
	Skin swab		
	Swab from abscess		
	Vesicula fluid		
	Insertion site—swab		
	Swab		
		Anus	If type = swab
		Perineum	If type = swab
Culture urinary tract/genitalia	Urine		
	Urine from catheter		
	Intrauterine device		
	Sperm		
	Urethra and cervix—swab		
		Cervix	If test category = swab, pus, secretion, tissue, or non-specific
		Genitalia externa	
		Uterus	
		Vagina	
		Catheter (nephrostomia)	
		Urethra	
Culture other	Tracheal secretion		
	Sputum, induced		
	Sputum		
	Nasopharynx secretion		
		Cavum oris	If test category = swab, pus, secretion, tissue, or type indicated in the text
		Tooth	
		Tounge	
		Nose	
		Sinus maxillaris	
		Throat/tonsil	
		Peritonsillar	
		Tracheostomia	
			
	Synovial fluid		
	Moucus sac		
	Fluid		
	Fistula—swab		
	Catheter apex		
	Pus		
	Secretion		
	Tissue		
	Faeces		
	Type indicated in text		
		Anatomical localisation indicated in text	
		Pilonidal cyst	If type = swab
		Rectum	If type = swab
		Eye	If test category = swab, pus, secretion, tissue, or type indicated in text
		Ear	
Respiratory tract bacteria PCR	*Bordetella pertussis*		
	*Mycoplasma pneumoniae*		
	*Chlamydia pneumoniae*		
	*Chlamydia ptsittici*		
	Legionella		
*Chlamydia trachomatis*	*Chlamydia trachomatis*		

Point-of-care tests.

**Table:**

Group	Type (reimbursement code)	Type (text)	Site
CRP	807120	CRP test	General practice
Strep-A	807109	Rapid antigen test for group A Streptococcus	General practice
POC urine tests	807101	Urine dip stick	General practice
	807122	Urine microscopy	General practice
	807105	Urine culture	General practice
	807189	Urine susceptibility analysis	General practice
Microscopy other	807116	Microscopy other material	General practice
CRP	837120	CRP test	OOHS
Strep-A	837109	Rapid antigen test for group A Streptococcus	OOHS
POC urine tests	837101	Urine dip stick	OOHS
	837122	Urine microscopy	OOHS
	837105	Urine culture	OOHS
	837189	Urine susceptibility analysis	OOHS
Microscopy other	837116	Microscopy other material	OOHS

## Appendix 2

Number of redeemed antibiotic courses from general practice stratified on type of antibiotics and year.

**Table:**

	2013		2014		2015		2016		2017	
	*n* (%)	Courses per 1000 inh. per year	*n* (%)	Courses per 1000 inh. per year	*n* (%)	Courses per 1000 inh. per year	*n* (%)	Courses per 1000 inh. per year	*n* (%)	Courses per 1000 inh. per year
Total antibiotic prescriptions	175,142 (100.0)	390.8	163,515 (100.0)	364.2	154,694 (100.0)	343.2	145,137 (100.0)	320.0	144,764 (100.0)	317.5
J01CE Beta-lactamase susceptible penicillins	60,757 (34.7)	135.6	56,748 (34.7)	126.4	51,544 (33.3)	114.4	45,660 (31.5)	100.7	47,625 (32.9)	104.5
J01CA Penicillins with extended spectrum	39,769 (22.7)	88.7	38,879 (23.8)	86.6	38,621 (25.0)	85.7	36,796 (25.4)	81.1	40,759 (28.2)	89.4
J01CF Beta-lactamase resistant penicillins	11,659 (6.7)	26.0	11,540 (7.1)	25.7	11,476 (7.4)	25.5	11,388 (7.8)	25.1	11,838 (8.2)	26.0
J01CR Combinations of penicillins, including beta-lactamase inhibitors	7,413 (4.2)	16.5	7,355 (4.5)	16.4	7,938 (5.1)	17.6	7,678 (5.3)	16.9	5,847 (4.0)	12.8
J01FA Macrolides	22,954 (13.1)	51.2	19,606 (12.0)	43.7	18,687 (12.1)	41.5	18,662 (12.9)	41.2	16,227 (11.2)	35.6
J01AA Tetracyclines	6,314 (3.6)	14.1	5,257 (3.2)	11.7	4,300 (2.8)	9.5	4,180 (2.9)	9.2	3,981 (2.7)	8.7
J01E Sulphonamides and trimethoprim	13,705 (7.8)	30.6	12,763 (7.8)	28.4	11,152 (7.2)	24.7	10,187 (7.0)	22.5	9,703 (6.7)	21.3
J01MA Fluoroquinolones	4748 (2.7)	10.6	4233 (2.6)	9.4	4074 (2.6)	9.0	3956 (2.7)	8.7	3503 (2.4)	7.7
P01AB01 Nitroimidazole derivatives	3,390 (1.9)	7.6	3,148 (1.9)	7.0	2,977 (1.9)	6.6	2,779 (1.9)	6.1	2,686 (1.9)	5.9
Other antibacterials	4,433 (2.5)	9.9	3,986 (2.4)	8.9	3,925 (2.5)	8.7	3,851 (2.7)	8.5	2,595 (1.8)	5.7
